# The Correlation between Pro- and Anti-Inflammatory Cytokines in Chronic Subdural Hematoma Patients Assessed with Factor Analysis

**DOI:** 10.1371/journal.pone.0090149

**Published:** 2014-02-27

**Authors:** Are Hugo Pripp, Milo Stanišić

**Affiliations:** 1 Department of Biostatistics, Epidemiology and Health Economics, Oslo University Hospital, Oslo, Norway; 2 Department of Neurosurgery, Oslo University Hospital, Oslo, Norway; Technische Universität Dresden, Medical Faculty, Germany

## Abstract

Chronic subdural hematoma (CSDH) is a relatively common disorder in neurosurgery on elderly patients, though the mechanism that causes the disease remains unclear. Studies have suggested that local anticoagulation and inflammatory changes may be important in its pathogenesis. Most studies have used a basic bivariate statistical analysis to assess complex immunological responses in patients with this disorder, hence a more sophisticated multivariate statistical approach might be warranted. Our objective was to assess the association and correlation between the pro- and anti-inflammatory responses in a cohort of patients with chronic subdural hematoma (n = 57) using an exploratory and confirmatory factor analysis. Thirteen assigned pro-inflammatory (TNF-α, IL-1β, IL-2, IL-2R, IL-6, IL-7, IL-12, IL-15, IL-17, CCL2, CXCL8, CXCL9 and CXCL10) and five assigned anti-inflammatory (IL-1RA, IL-4, IL-5, IL-10 and IL-13) cytokines from blood and hematoma fluid samples were examined. Exploratory factor analysis indicated two major underlying immunological processes expressed by the cytokines in both blood and hematoma fluid, but with a different pattern and particularly regarding the cytokines IL-13, IL-6, IL-4 and TNF-α. Scores from confirmatory factor analysis models exhibited a higher correlation between pro- and anti-inflammatory activities in blood (r  = 0.98) than in hematoma fluid samples (r  = 0.92). However, correlations of inflammatory processes between blood and hematoma fluid samples were lower and non-significant. A structural equation model showed a significant association between increased anti-inflammatory activity in hematoma fluid samples and a lower risk of recurrence, but this relationship was not statistically significant in venous blood samples. Moreover, these findings indicate that anti-inflammatory activities in the hematoma may play a role in the risk of a recurrence of CSDH.

## Introduction

Chronic subdural hematoma (CSDH) is a relatively common disorder seen in neurosurgery on elderly patients, and often associated with a head trauma. It is clinically recognized by a clearly bounded accumulation of blood, blood breakdown products and plasma exudate between the dura mater and the arachnoid [1]–[4]. The content of CSDH is encapsulated by granulation tissue containing newly formed blood vessels, inflammatory cells and proliferating fibroblasts [5].

Several studies have suggested that local inflammatory reaction and local abnormality in coagulo-fibrinolytic system are pathogenic processes underlying CSDH development [6]–[11]. Consequently, inflammatory cytokines as indicators of local inflammation have been investigated to elucidate the pathogenic mechanism that causes the disease and the possibility to develop adjuvant non-surgical therapeutic alternatives [12]–[15]. In accordance with a supposed role of inflammation in the development of CSDH, corticosteroids have been used [16], and there is a reported case of treatment with the tumor necrosis factor (TNF)-alpha inhibitor drug, infliximab [17]. However, the corresponding pro- and anti- inflammatory responses and their associations in both the blood and the hematoma of patients with CSDH has not been fully established and understood.

In recent studies on immunological responses of a Norwegian cohort of CSDH patients [14], [15], the authors used descriptive statistics (e.g. mean, median and standard deviation, etc.) and tests (e.g. t-tests and Wilcoxon ranked sign tests) to assess selected pro- and anti-inflammatory cytokines in venous blood and hematoma fluid samples to elucidate the biological processes underlying this disorder. They found that the immune responses occurred both locally in the hematoma fluid and systematically in the venous blood. It was an enhanced release of TNF-α, IL-1β, IL-2 and IL-4 in blood compared with values in hematoma fluid and an enhanced release of IL-2R, IL-5, IL-6, IL-7, IL-10, IL-13, CCL2, CXCL8, CXCL9 and CXCL10 in hematoma fluid compared with venous blood. Nonetheless, the large number of cytokines examined made it methodologically difficult to assess overall associations and correlations between immunological activities and their relation to clinical outcome such as, e.g. recurrence. Recurrence of CSDH is a major adverse postsurgical clinical outcome, which may result in repeated surgical treatments, risk of further complications and mortality.

From a biostatistical point of view, the analysis of cytokine data often raises several challenging issues. On the one hand, the variables can be highly skewed, with missing observations scattered throughout the dataset, while on the other, levels of different cytokines can be highly correlated and closely related to similar underlying biological process. Genser et al. [18] reviewed the use of classical bivariate and multivariate statistical techniques (e.g. factor analysis and cluster analysis) as well as more advanced methods such as path analysis and structural equation modeling in their guide to the statistical analysis of immunological data. They found that most immunological studies use rather basic statistical methods on immunological data, even when there are many relationships between the study variables. Thus, a more advanced and sophisticated use of multivariate statistical techniques to assess complex immunological data is recommended.

The biological actions of the cytokines can generally be considered as either pro- or anti-inflammatory. Therefore, the multivariate statistical method factor analysis may be suited to assess the association and correlation between pro- or anti-inflammatory activities expressed biologically by the cytokines. This statistical method is usually divided into an exploratory factor analysis and confirmatory factor analysis. An exploratory factor analysis is a variable reduction technique that seeks to identify the number of latent constructs or common factors, i.e. a variable not measured directly, of a set of measured variables. Observed variables are then statistically assumed to be a linear combination of the underlying factors and random error. A confirmatory factor analysis is a related, but hypothesis-driven approach developed to assess whether associations among many variables confirm one or several hypothesized underlying factors [19], [20]. Both an exploratory- and confirmatory factor analysis have been used successfully in the analysis of cytokines in medical research [21]–[24], but to the best of our knowledge not on immunological characteristics in CSDH patients.

Cytokine data from a Norwegian cohort of CSDH patients [14], [15] without other factors that could strongly confound the levels of cytokines were analyzed with both an exploratory and confirmatory factor analysis to assess the associations between the pro- and anti-immunological responses in venous blood and hematoma fluid samples. It was then possible to quantify the correlation between the latent pro- or anti-inflammatory activities biologically expressed by numerous cytokines and their association with postsurgical recurrence of CSDH.

## Materials and Methods

### Ethics Statement

The Regional Ethical Committee of the Health Region of Southeast Norway (S-06281a) approved the study of human subjects, and the Norwegian Directorate of Health approved the establishment of a research bio-bank. Furthermore, written informed consent was obtained from the patients or their significant others before study inclusion.

### Patient Population

Patients with the following features were excluded from the statistical analyses: vp-shunt, postcraniotomy, infective, inflammatory, hematological and neoplastic diseases, liver dysfunction, dementia, brain infarct and current use of anti-inflammatory therapy. These were excluded to assess a “healthy” cohort, in which cytokine levels were not confounded by other factors and, thus, assumed to be specifically related to CSDH.

### Statistical Analysis

The concentrations of cytokines (after logarithmic transformation) are described using mean (standard deviation), while statistical assumptions regarding normal distribution were assessed with boxplots and histograms. The mean difference between venous blood and hematoma fluid concentration was assessed with a paired sample t-test, both with and without a Bonferroni correction for multiple testing.

Since previous studies of this cohort have strongly hypothesized that the immunological processes and expression of cytokines evolve differently in venous blood versus hematoma fluid [14], [15], both exploratory and confirmatory factor analyses were conducted with separate models for venous blood and hematoma fluid samples. An exploratory factor analysis was conducted according to the default options in the software, with all (logarithmic transformed) variables treated as continuous, and loadings were extracted using maximum likelihood and rotation performed with the oblique geomin method. The number of extracted factors was selected by assessing screeplots and model fit statistics, i.e. Root Mean Square Error of Approximation (RMSEA), Comparative Fit Index (CFI) and Tucker and Lewis Index (TLI). Using statistical notation, a factor model postulates that the observed variables X_1_, X_2_, …,X_p_ (i.e. concentration of the individual cytokines ) with means μ_1_, μ_1_, …, µ_p_ are linearly dependent upon a few unobservable random variables F_1_, F_2_, …, F_m_ called common factors, and p additional sources of variation ε_1_, ε_2_, …, ε_p_ called errors. Hence, the exploratory factor analysis model is generally given as Equation 1:
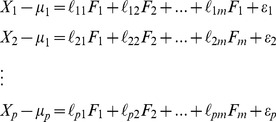
(1)


The coefficient *l*_ij_ is then the loading of the *i*th variable on the *j*th factor.

A confirmative factor analysis was conducted based on assigned pro-inflammatory (i.e. IL-1β, IL-2, IL-7, IL-6, CCL2, CXCL9, TNF-α, IL-12, IL-2R, IL-15, CXCL10, IL-17 and CXCL8) or anti-inflammatory (i.e. IL-4, IL-5, IL-10, IL-1RA and IL-13) cytokines. As a result, pro- or anti-inflammatory activity would be the hypothesized underlying factors in a confirmatory factor analysis model. Using statistical notation, the confirmatory factor analysis model is given in Equation 2 with an assumed pro-inflammatory common factor F_pro_ and anti-inflammatory common factor F_anti_:
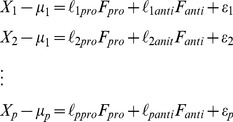
(2)


The coefficients *l*_ipro_ and *l*_ianti_ are the loading of the *i*th variable. For designated pro-inflammatory cytokines, the coefficient *l*_ianti_ is set to 0, while correspondingly, for designated anti-inflammatory cytokines, the coefficient *l*_ipro_ is set to 0 in the confirmatory factor model.

A model fit of the factor analysis models was done assessing the p-values, the loadings, the amount of variance explained and the above-mentioned model fit statistics. Variables with non-significant standardized loading values in confirmatory factor analysis models were excluded to reduce the number of estimated parameters.

A structural equation model on the relationship between anti-inflammatory activity in hematoma fluid and venous blood on the risk of recurrence of CSDH were then estimated. The standardized coefficients in these models were estimated using maximum likelihood.

All statistical analyses for exploratory and confirmatory factor analysis were performed with Mplus version 7.0 (Muthen & Muthen, Los Angeles, CA, USA), and PASW Statistics 18 (IBM Corporation, Armonk, New York, USA) was used for data management and descriptive statistics. The statistical significance level was set to p<0.05.

## Results

Descriptive statistics of the individual cytokines are shown using mean (standard deviation) concentration of cytokines after logarithmic transformation (log pg/ml) in venous blood and hematoma fluid. The mean differences between the concentration of cytokines in venous blood and hematoma fluid are given with a 95% confidence interval and p-values, as well as with statistical tests after Bonferroni correction (Table 1). The data distributions examined recommended the use of parametric tests instead of non-parametric tests [25], and there was no statistically significant difference between venous blood and hematoma fluid concentration for cytokines IL-1β, IL-2, IL-12, IL-15 and IL-1RA, a significantly higher concentration in venous blood compared to hematoma fluid for TNF-α and a significantly higher concentration in hematoma fluid compared to venous blood for IL-7, IL-6, CCL2, CXCL9, IL-2R, CXCL10, IL-17, CXCL8, IL-5, IL-10 and IL-13. The difference was also statistically significant after Bonferroni correction (except for IL-17).

**Table 1:** Concentration of cytokines (log pg/ml) in venous blood and hematoma fluid samples.

Cytokine	Venous blood		Hematoma fluid		N	Mean difference
	N	Mean (SD)	N	Mean (SD)		(95% CI), p-value
Pro-inflammatory cytokines						
IL-1β	51	1.4 (0.7)	43	1.3 (0.7)	41	0.2 (0.0–0.4), 0.082
IL-2	50	0.8 (0.8)	45	0.7 (0.5)	45	0.2 (0.0–0.4), 0.113
IL-7	55	1.5 (0.3)	56	1.7 (0.2)	54	−0.3 (−0.3–−0.2), <0.001^B^
IL-6	41	1.1 (0.8)	41	3.8 (0.9)	31	−2.8 (−3.2–−2.4), <0.001^B^
CCL2 (MCP-1)	56	2.3 (0.4)	53	3.5 (0.5)	52	−1.2 (−1.4–−1.0), <0.001^B^
CXCL9 (MIG)	55	1.5 (0.4)	54	1.9 (0.6)	53	−0.3 (−0.5–−0.1), 0.001^B^
TNF-α	41	0.6 (0.5)	38	0.5 (0.5)	36	0.2 (0.1–0.3), 0.002^B^
IL-12	57	2.0 (0.3)	56	1.9 (0.3)	56	0.0 (−0.1–0.2), 0.394
IL-2R	57	2.4 (0.3)	56	2.8 (0.3)	56	−0.3 (−0.5–−0.2), <0.001^B^
IL-15	57	1.8 (0.4)	56	1.9 (0.4)	56	−0.1 (−0.2–0.0), 0.157
CXCL10 (IP-10)	57	1.6 (0.4)	51	2.5 (0.5)	51	−0.9 (−1.0–−0.8), <0.001^B^
IL-17	29	1.2 (0.7)	45	1.3 (0.6)	27	−0.3 (−0.6–0.0), 0.031
CXCL8 (IL-8)	52	0.9 (0.5)	56	3.3 (0.9)	51	−2.5 (−2.8–−2.2), <0.001^B^
Anti-inflammatory cytokines						
IL-4	57	1.5 (0.5)	56	1.1 (0.4)	56	0.4 (0.3–0.5), 0.001^B^
IL-5	40	1.0 (0.4)	50	1.4 (0.6)	38	−0.4 (−0.6–−0.1), 0.003
IL-10	57	1.0 (0.5)	56	1.4 (0.4)	56	−0.3 (−0.5–−0.2), <0.001^B^
IL-1RA	57	2.7 (0.5)	56	2.7 (0.5)	56	0.0 (−0.1–0.2), 0.522
IL-13	43	0.9 (0.5)	53	1.4 (0.5)	43	−0.4 (−0.7–−0.2), <0.001^B^

B) Statistically significant after Bonferroni correction of multiple testing.

### Exploratory Factor Analysis

Two factors were extracted from a separate statistical analysis of the blood and hematoma fluid samples. Model fit statistics and the loading values after oblique rotation are shown in Table 2. The loading values in Table 2 correspond to the coefficients *l* in Equation 1 if expressed using a statistical notation of the factor model, e.g. a concentration of IL-1β would correspond to X_1_, and a loading value of 0.76 would correspond to the coefficient l_11_ for Factor 1 (F1 in Equation 1). Due to some patients with missing data on multiple cytokines, 51 and 50 patients were included with blood and hematoma fluid samples, respectively.

**Table 2:** Rotated standardized loadings from exploratory factor analysis.

Cytokine	Venous blood (n = 51)		Hematoma fluid (n = 50)	
	Factor 1	Factor 2	Factor 1	Factor 2
Pro-inflammatory cytokines				
IL-1β	**0.76**	0.10	**0.78**	−0.18
IL-2	**0.74**	0.10	**0.52**	−0.13
IL-7	**0.93**	−0.15	**0.63**	−0.02
IL-6	**0.53**	−0.19	0.16	**0.49**
CCL2 (MCP-1)	**0.79**	0.00	**0.48**	−0.28
CXCL9 (MIG)	**0.78**	0.20	**0.49**	0.30
TNF-α	**0.50**	**0.44**	**0.78**	−0.01
IL-12	**0.51**	**0.58**	**0.80**	0.23
IL-2R	0.34	**0.65**	−0.05	**0.77**
IL-15	**0.47**	**0.67**	0.25	0.38
CXCL10 (IP-10)	−0.33	**0.73**	0.08	**0.58**
IL-17	**0.44**	−0.11	0.19	0.03
CXCL8 (IL-8)	0.21	−0.04	0.32	0.16
Anti-inflammatory cytokines				
IL-4	0.00	**0.90**	0.36	0.38
IL-5	−0.09	**0.48**	−0.06	**0.88**
IL-10	0.24	**0.74**	0.36	**0.67**
IL-1RA	**0.75**	0.33	**0.89**	0.22
IL-13	**0.81**	−0.12	0.01	**0.79**
Model fit statistics				
Eigenvalue	8.69	2.39	6.20	2.76
Percentageexplained	48.3	13.3	34.4	15.3
RMSEA (90%CI)	0.211 (0.188–0.235)		0.243 (0.220–0.266)	
CFI	0.704		0.486	
TLI	0.616		0.334	

Bold letters indicate statistically significant (p<0.05) standardized loadings with an absolute value above 0.4.

In both the blood and hematoma fluid samples, CXCL8 did not have significant loading values above 0.4. The pro-inflammatory cytokines IL-1β, IL-2, IL-7, IL-6, CCL2, CXCL9, TNF-α, IL-12, IL-15, IL-17 and the anti-inflammatory IL-1RA and IL-13 were related to the first factor, and the pro-inflammatory cytokines TNF-α, IL-12, IL-2R, IL-15 and CXCL10 and the anti-inflammatory IL-4, IL-5 and IL-10 to the second extracted factor from blood samples. From the hematoma fluid samples, the pro-inflammatory cytokines IL-1β, IL-2, IL-7, CCL2, CXCL9, TNF-α and IL-12, together with the anti-inflammatory IL-1RA, were related strongly to the first factor, while the pro-inflammatory cytokines IL-6, IL-2R and CXCL10, together with the anti-inflammatory IL-5, IL-10 and IL-13, were strongly related to the second factor. In particular, the cytokines IL-13, IL-6, IL-4 and TNF- α exhibited differences in loadings between an exploratory factor analysis from blood and hematoma samples.

### Confirmatory Factor Analysis

A confirmatory factor analysis with cytokines assigned to pro- or anti-inflammatory biological actions was conducted, with standardized loading values from a separate confirmatory factor analysis of venous blood and hematoma samples with model fit statistics shown in Table 3. The IL-6 and CXCL8 in blood samples and CCL2, IL-5 and IL-13 in hematoma samples did not have significant standardized loadings, and were therefore excluded from the statistical models. The loading values in Table 3 correspond to the coefficients *l* in Equation 2 when expressed using a statistical notation of the confirmatory factor model. Thus, a concentration of IL-1β would correspond to X_1_, and the loading value of 0.73 would correspond to the coefficient l_1pro_ of the common pro-inflammatory activity factor (F_pro_ in Equation 2).

**Table 3:** Standardized loadings from confirmatory factor analysis.

Cytokine	Venous blood (n = 51)	Hematoma fluid (n = 50)
Pro-inflammatory activity as a latent variable		
IL-1β	**0.73**	**0.66**
IL-2	**0.71**	**0.41**
IL-7	**0.65**	**0.52**
IL-6	–	0.30
CCL2 (MCP-1)	**0.67**	–
CXCL9 (MIG)	**0.82**	**0.57**
TNF-α	**0.81**	**0.75**
IL-12	**0.92**	**0.85**
IL-2R	**0.84**	**0.37**
IL-15	**0.94**	**0.46**
CXCL10 (IP-10)	0.30	**0.47**
IL-17	0.26	**0.17**
CXCL8 (IL-8)	–	0.35
Anti-inflammatory activity as a latent variable		
IL-4	**0.71**	**0.51**
IL-5	**0.32**	–
IL-10	**0.78**	**0.66**
IL-1RA	**0.89**	**0.92**
IL-13	**0.59**	–
Model fit statistics		
RMSEA (90%CI)	0.226 (0.201–0.251)	0.235 (0.208–0.262)
CFI	0.660	0.505
TLI	0.603	0.416

Bold letters indicate statistically significant (p<0.05) standardized loadings with an absolute value above 0.4. Cytokines with non-significant loadings were excluded from the final model.

Factor scores to these latent variables from a confirmative factor analysis were estimated for each patient, which is a relative value to the specific latent variable (i.e. pro or anti-inflammatory activity given in statistical notation as either F_pro_ or F_anti_ in Equation 2) for a given patient based on the confirmatory factor analysis model. By default in the Mplus software, the estimated factor scores have a mean of zero. Thus, factor scores cannot be interpreted in absolute terms, e.g. a factor score of 0 does not indicate an absence of inflammatory activity, while a negative factor score does not indicate a negative inflammatory activity in biological terms. Based on our statistical model, a patient with a high factor score on pro-inflammatory activity in systemic samples is therefore assumed to have a high relative pro-inflammatory activity in the systemic sample compared with the other patients.

**Figure 1:**
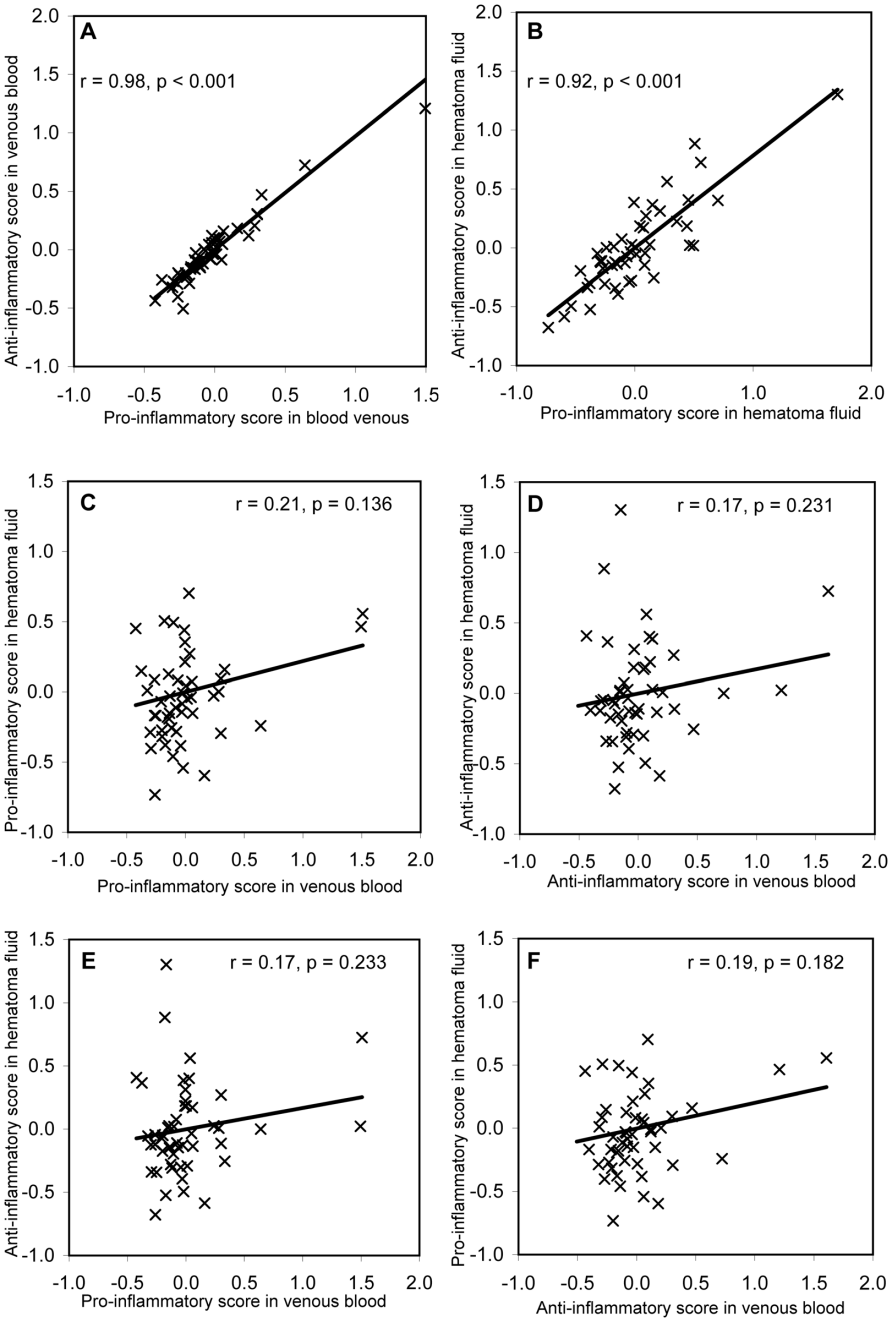
The correlation between the factor scores from the confirmatory factor analysis models in Table 3. These scores are the relative values of the underlying factors assumed to express pro- or anti-inflammatory activity in venous blood or hematoma fluid samples. They are statistically standardized to a mean of zero. The Pearson correlation coefficients and p-values are stated in the corresponding plots.

The correlations between underlying pro- and anti-inflammatory activities in both blood and hematoma fluid samples could then be quantified using these factor scores, with plots of the relationship between factor scores with corresponding Pearson correlation coefficients shown in Figure 1. A very high correlation was found between the factor scores for pro- versus anti-inflammatory activity in venous blood samples (r  = 0.98, Figure 1A) and lower, but was still highly significant in hematoma fluid samples (r  = 0.92, Figure 1B). Substantially lower and non-significant correlations were found between factor scores for pro-inflammatory activity in venous blood versus hematoma fluid samples (r  = 0.21, Figure 1C) or anti-inflammatory activity in venous blood versus hematoma fluid samples (r  = 0.17, Figure 1D). The correlations between factor score of pro-inflammatory in venous blood versus anti-inflammatory in hematoma fluid samples (r  = 0.17, Figure 1E), as well as between anti-inflammatory in venous blood versus pro-inflammatory in hematoma samples (r  = 19, Figure 1F) were both low and non-significant.

### Structure Equation Model on Anti-inflammatory Activity and Risk of Recurrence

**Figure 2:**
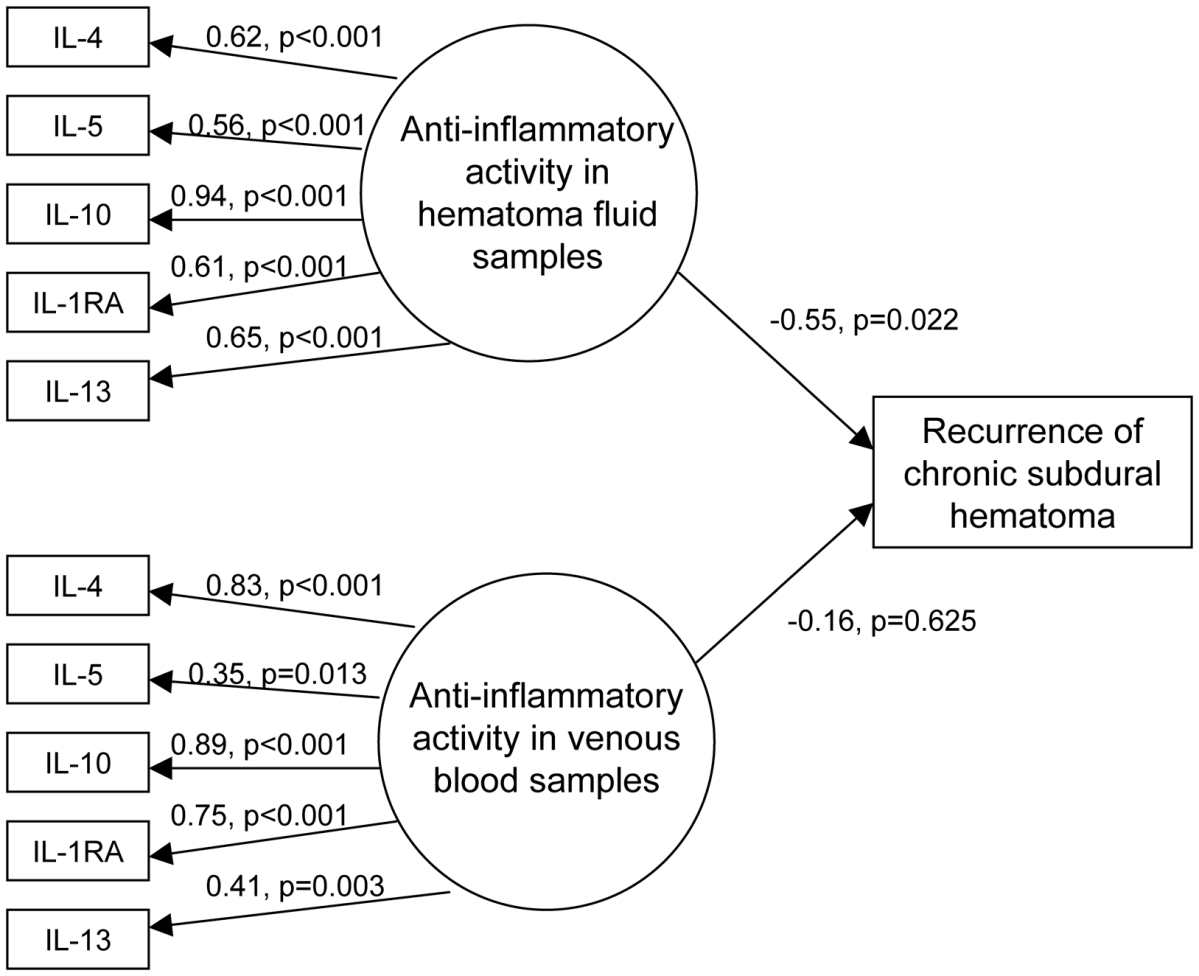
Structural equation model on relationship between anti-inflammatory activity and risk of recurrence.

A structural equation model on anti-inflammatory activity in both hematoma fluid and venous blood samples in relation to risk of recurrence (n = 56 of which seven patients had recurrence) is presented in Figure 2. All anti-inflammatory cytokines had statistically significant standardized loadings to the respective underlying factors expressing anti-inflammatory activity in hematoma fluid samples and in venous blood samples. The standardized coefficients for risk of recurrence were negative for both anti-inflammatory activity in venous blood and hematoma fluid, but only statistically significant in hematoma fluid samples.

## Discussion

### Cytokine Patterns

Table 1 shows descriptive statistics on the concentration of cytokines in venous blood and hematoma fluid samples and their mean differences. Only samples with data in both venous blood and hematoma fluid could be assessed with a paired sample t-test, which is reflected by the differences in the number of samples analyzed. Basic descriptive statistics of both venous blood and hematoma fluids samples from this cohort using data before logarithmic transformation and the comparisons with paired sample t-tests or Wilcoxon signed rank tests have been reported elsewhere [14], [15]. Logarithmic transformation was applied to make the data more normally distributed, and provided data on where parametric tests would be recommended instead of non-parametric tests. Generally speaking, there were higher concentrations of both pro-inflammatory and anti-inflammatory cytokines in hematoma fluid compared with venous blood samples.

Our underlying hypothesis was that the levels of cytokines developed with different mechanisms systematically in venous blood and locally in hematoma fluid [14], [15]. Therefore, both the exploratory- and confirmatory factor analysis models were separately developed for venous blood and hematoma fluid. Results from the two exploratory factor analysis models (Table 2) indicated a different underlying cytokine pattern in venous blood compared with hematoma fluid samples, which seemed especially so for the pro-inflammatory cytokines IL-6 and TNF-α and the anti-inflammatory cytokines IL-4 and IL-13. Since the cytokines IL-13, IL-6, IL-4 and TNF-α in particular displayed a different loading pattern in blood compared to hematoma fluid samples, they could play a different role in the local inflammatory response in the hematoma compared with the systemic response assessed in blood samples. Additionally, the pro-inflammatory cytokine TNF-α is considered to be a primary mediator of the inflammatory response to stimulate the synthesis and release of other cytokines [26].

The underlying pro- or anti-inflammatory biological actions are not directly measured as such, but biochemically expressed through the concentration of specific cytokines. However, from a statistical perspective, we can assume an unmeasured variable that expresses the latent pro- or anti-inflammatory activity. This unmeasured (or latent) variable is the factor. The interpretation of a standardized loading in the confirmatory factor analysis models can be understood as the correlation between an individual cytokine and this latent biological process. Using the model based on venous blood samples for illustration (Table 3), the standardized loading value between IL-1β and pro-inflammatory activity in blood (i.e. 0.73) is interpreted as the correlation between an unmeasured variable that expresses the latent pro-inflammatory activity and the concentration of IL-1β. This is generally stated using the statistical notation in Equation 2.

Estimated standardized loading values were mostly higher in blood than in hematoma fluid samples, which is also reflected by the partially better statistical model fit. The estimated factor scores from the confirmatory factor analysis clearly revealed a different correlation between inflammatory responses (Figure 1). A high correlation was seen between pro- and anti-inflammatory scores within venous blood and hematoma fluid samples, but was lower between venous blood and hematoma fluid samples. This is in accordance with a different immune response between blood and the CSDH region. A correlation and balance between pro- and anti-inflammatory markers as a response to infection is shown to be of general clinical importance [27], [28], and our results from confirmatory factor analysis showed that the inflammatory response as expressed by cytokine levels in blood samples was not reflected in the hematoma fluid samples.

### Inflammatory Activity and Risk of Recurrence

Postoperative recurrence is an unwanted clinical outcome after surgery on CSDH patients, and recurrence with corresponding surgical treatment is a major adverse clinical outcome [2]. Using basic statistical approaches, its relationship with cytokine concentration has been inconclusive using data from this cohort [14], [15]. Since anti- and pro-inflammatory activity was strongly correlated within hematoma fluid or venous blood samples, only the anti-inflammatory activity was considered in the structural equation model on risk of recurrence to help avoid multicollinearity issues (Figure 2). A reduced risk of recurrence with increasing anti-inflammatory activity was only statistically significant in hematoma blood samples. Frati et al. [29] found increased CSDH levels of pro-inflammatory cytokines IL-6 and IL-8 in patients where recurrence occurred, thereby suggesting a prolonged postoperative anti-inflammatory medicine given as prophylaxis to prevent a recurrence of CSDH. Our analyses based on a structural equation modeling approach support an association between anti-inflammatory activity in hematoma and a reduced risk of recurrence. This biostatistical approach has been shown to be useful in assessing the relationship between inflammation and clinical characteristics in fields besides CSDH [30], [31].

### A Multivariate Biostatistical Approach to the Analysis of Cytokines

Multivariate statistics involve methods that simultaneously conduct an analysis of more than one (dependent) variable, and include methods such as, e.g. multivariate analysis of variance, principal component analysis, factor analysis and artificial neural networks. Multivariate statistical methods are frequently applied in several medical disciplines, as well as in engineering and chemometrics, which could provide insight when applied to immunological data [18]. To the best of our knowledge, the application of multivariate statistical methods is limited in the field of CSDH, and a literature search on “chronic subdural hematoma” and common multivariate statistical methods identified only one study [32]. That study applied an artificial neural network to predict the outcome from CSDH, and found that artificial neural networks displayed a better performance compared to the regression model.

It is generally recommend that a factor analysis model obtain fit statistics of RMSEA <0.06, CFI >0.95 or TLI >0.95 to indicate a substantial model fit [33]. The effect of logarithmic transformation and the presence of missing data may have an influence on the model fit, but most likely the assessment of cytokines as only either pro- or anti-inflammatory does not completely reflect all the complex immunological responses ascribed to the cytokine system [34]. Furthermore, our sample to variable ratio was also on the lower side. However, this approach demonstrated that the systemic innate immune responses in blood were different from the local responses at the site of CSDH, and that increased anti-inflammatory activity expressed through elevated levels of selected cytokines in hematoma fluid may be related to a reduced risk of recurrence after surgery.
